# Standalone Transoral Robotic Surgery for Obstructive Sleep Apnoea: A Systematic Literature Review of Clinical Outcomes

**DOI:** 10.3390/life16020332

**Published:** 2026-02-14

**Authors:** Konstantinos Chaidas, Stavroula Mouratidou

**Affiliations:** 1 Ear, Nose, and Throat Department, Democritus University of Thrace—Medical School, 68100 Alexandroupolis, Greece; 2 Ear, Nose, and Throat Department, Imperial College Healthcare NHS Trust, London W2 1NY, UK; 3 Otolaryngology—Head and Neck Department, Guy’s and St Thomas’ NHS Foundation Trust, London SE1 9RT, UK; stavroulamouratidou@gmail.com

**Keywords:** TORS, OSA, obstructive sleep apnoea, TORS BOT, transoral robotic surgery, base of tongue surgery

## Abstract

Transoral robotic surgery (TORS) offers a targeted surgical option for addressing base of tongue (BOT) and epiglottic obstruction in selected obstructive sleep apnoea (OSA) cases; however, most published evidence evaluates TORS within multilevel approaches, limiting understanding of single-level outcomes. A PRISMA-guided systematic review of PubMed, Embase, and Central Cochrane was conducted from inception to March 2025, aiming to evaluate objective sleep outcomes and patient-reported measures following single-level TORS BOT surgery. Inclusion criteria were adult patients with moderate-to-severe OSA and CPAP failure/intolerance, with evidence of BOT hypertrophy. Of 219 screened records, five studies met the inclusion criteria with 105 patients. Eighty-six (81.9%) were male with a combined mean age of 45.2 years and BMI of 28.2 kg/m^2^. Combined mean AHI improved from 34.2 preoperatively to 14.7 events/hour postoperatively. Reported surgical success ranged from 54.2% to 100%. Where reported, ESS improved postoperatively with a combined mean reduction from 13 to 4.5. Most commonly reported complications were dysgeusia (n = 16, 15.2%), dysphagia/odynophagia (n = 14, 13.3%), and postoperative bleeding (n = 10, 9.5%). Single-level TORS BOT appears to improve objective and subjective outcomes in carefully selected patients, although heterogeneity and inconsistency of reported outcomes limit definitive conclusions and highlight the need for standardised outcome reporting and follow-up.

## 1. Introduction

Untreated obstructive sleep apnoea (OSA) has been consistently associated with severe cardiovascular morbidity and impaired quality of life. Continuous positive airway pressure (CPAP) remains the mainstay of treatment, with recent studies concluding that CPAP use for at least four hours per night has been associated with significant morbidity risk reduction and improved outcomes [1]. However, long-term adherence is frequently limited by intolerance, while a proportion of patients continue to experience persistent symptoms or inadequate control despite CPAP.

Upper airway surgery has been utilised as an alternative treatment [2], referring to a broad spectrum of procedures targeting different anatomical levels of obstruction, and has been frequently performed as a multilevel approach. In recent years, drug-induced sedation endoscopy (DISE) has refined surgical decision-making by enabling dynamic characterisation of the pattern and level of airway collapse, aiding in a focused and personalised surgical approach in selected cases [3]. In patients with tongue base hypertrophy and collapse, several surgical options exist, ranging from endoscopic approaches and hypoglossal nerve stimulation devices to transoral robotic surgery (TORS).

TORS has emerged as an effective surgical option in patients with DISE evidence of base of tongue (BOT) hypertrophy, with or without epiglottic obstruction, since its first application in 2010 [4]. Even though current evidence has reported improvements in objective sleep parameters and symptom burden, most studies evaluate TORS as part of a multilevel surgical approach, limiting the ability to isolate its objective outcomes and provide high-quality data. Interpretation is further constrained by inconsistency in reporting surgical success, heterogeneous follow-up intervals and limited disease-specific patient-reported outcome measures. As a result, previously published systematic reviews and meta-analyses included multilevel approaches, providing limited clarity regarding the expected benefit of the single-level TORS BOT procedure [5].

This systematic review aims to address the gap in current knowledge regarding sleep-related objective outcomes and subjective quality of life outcomes following single-level TORS addressing BOT and/or epiglottic obstruction for selected cases with moderate-severe OSA and CPAP failure/intolerance.

## 2. Materials and Methods

### 2.1. Literature Search Strategy

A systematic review was conducted in accordance with the PRISMA guidelines [6]. PubMed (MEDLINE), EMBASE and CENTRAL (Central Cochrane Library) were systematically searched from inception up to and including March 2025. Predefined search terms using Medical Subject Headings (MeSH) were utilised relating to TORS, tongue base/epiglottic procedures, and OSA. Additionally, reference lists of enrolled studies were screened manually to identify additional eligible studies. All records were imported into Endnote for deduplication, review and citation management. Results were initially screened by title and abstract, and duplicated results were removed. Studies published in English were considered eligible.

### 2.2. Eligibility Criteria

Eligible studies included adult patients (age > 18 years) with moderate-to-severe OSA who underwent TORS tongue base and/or epiglottic surgery, performed as a single treatment modality. Inclusion criteria also included available pre- and post-operative polysomnography confirmed apnoea-hypopnea index (AHI) and clearly stated indications for TORS based on clinical examination and DISE. Exclusion criteria were insufficient data, TORS BOT being performed as part of a multilevel surgical approach, case reports and/or studies including <3 participants.

### 2.3. Definitions

Moderate to severe OSA was defined as AHI ≥ 15 episodes/hour. Surgical response/success was assessed with success defined as an AHI < 20 as well as >50% reduction in the pre-operative AHI, and cure rate as was defined as a post-operative AHI < 5 [7].

### 2.4. Data Extraction and Analysis

Two authors independently assessed study eligibility. Subsequently, data extraction was performed by the first author and the second author, verifying the collected data. Disagreements were addressed and resolved by consensus. Collected data included study design, patients’ demographics, surgical technique and follow-up duration. Primary outcomes of interest were success, cure and failure rates at 6- and 12 months as well as objective outcome measures, mainly polysomnography confirmed AHI. Secondary outcomes were subjective outcome measures, including Epworth Sleepiness Score (ESS) and snoring Visual Analogue Scale (VAS), volume of resected tissue, operating room times, postoperative complications, and need for revision surgery. Given the heterogeneity in study design, follow-up timing and outcome definitions, meta-analysis was not performed. Where studies reported means and standard deviations for the same outcome at comparable follow-up timepoints, combined means were calculated from available data weighted by sample size to facilitate summary reporting. These values were intended for descriptive purposes, were not polled effect sizes and should not be interpreted as meta-analytic estimates, while most findings were summarised narratively.

### 2.5. Study Quality Assessment

Risk of bias was assessed independently by two authors. The ROBINS-I V2 tool [8] was utilised for non-randomised studies, and discrepancies were resolved by consensus.

## 3. Results

### 3.1. Study and Patient’s Characteristics

**Figure 1:**
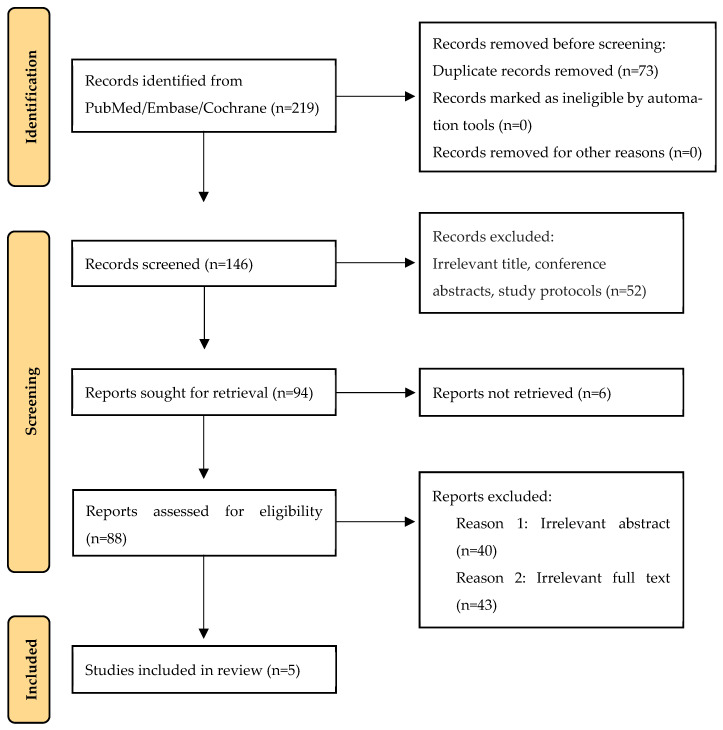
The literature search and article selection; n: number of studies.

A total of 219 records were screened (Figure 1), and five studies met the inclusion criteria, including 105 patients who underwent single-level TORS BOT with or without concurrent epiglottic surgery (Figure 1). Four studies were prospective, and one study had a retrospective design, as shown in Table 1. Overall, 86 patients were male (81.9%) with a sample-size-weighted combined mean age of 45.2 years (range 30–64) and a combined mean body mass index (BMI) of 28.2 kg/m^2^ (range 18–32).

Across included studies, eligibility criteria consistently included moderate-to-severe OSA confirmed by polysomnography, intolerance or failure of CPAP and/or mandibular advancement device, BMI < 35 kg/m^2^ and DISE findings confirming predominant BOT obstruction with or without epiglottic collapse. Follow-up reporting was heterogeneous, with a mean duration reported in two studies (12 to 18.9 ± 6.2 months), and the minimum follow-up ranged from >1 to 12 months (Table 1).

### 3.2. Risk of Bias

**Figure 2:**
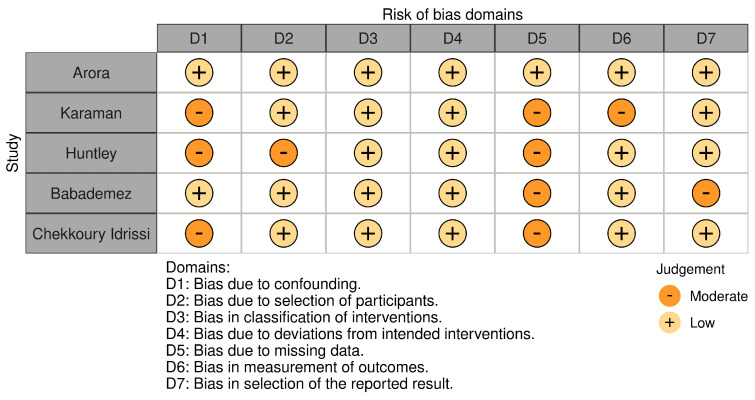
Risk of bias assessment tool.

All studies were classified as low to moderate risk, as shown in Figure 2. The most frequent concerns were related to confounding and missing outcome data. Moderate risk regarding bias of missing data was concluded for all but one study, deriving from a lack of consistent reporting of handling of missing data. Most studies were otherwise evaluated as low risk across most bias domains.

### 3.3. Surgical Characteristics

All included patients underwent TORS BOT surgery, while concurrent TORS epiglottoplasty (EGP) was performed in 10 patients (9.5%). Only one study excluded patients with prior tonsillar surgery, accounting for 37 patients (35.2%). Two studies provided intraoperative tongue base resection volumes, with a combined mean of 15 mL (range: 10–20 mL). Combined mean robotic set-up time was 23.6 min, and mean TORS TBR +/− EGP surgical time was 37.1 min (range: 20–63 min).

### 3.4. Objective Sleep Outcomes

All studies provided mean pre- and postoperative AHI (Table 2), with a combined mean pre-operative AHI of 34.2 reduced to 14.7 postoperatively. All studies followed Sher’s criteria to document surgical response/success and cure rates based on AHI reduction. Some authors include an ESS < 10 as part of their surgical response assessment; however, this is inconsistent in the literature. Surgical success ranged from 54.2% to 100%, with a weighted overall estimated rate of 66.4%. Only two studies evaluated their cure rates, ranging between 20.8 and 36.4%, with an overall weighted estimated surgical cure rate of 26.6%. Saturation outcomes were inconsistently reported as lowest oxygen saturation (LSAT) or mean SaO_2_ in two studies, as represented in Table 2.

### 3.5. Subjective Outcomes

Three studies reported ESS outcomes, with a combined mean ESS reduction from 13 preoperatively to 4.5 postoperatively. Other OSA and TORS-specific patient-reported outcome measures (PROMS) were variably reported and generally improved where measured (Table 3).

### 3.6. Postoperative Complications

All studies reported postoperative complications. The most commonly encountered complication was dysgeusia in 16 cases (15.2%), followed by dysphagia/odynophagia in 14 cases (13.3%), three of which (18.8%) were initially managed with NGT feeding. Bleeding was reported in 10 cases (9.5%), which was self-limited or resolved with conservative treatment in 9 cases (90%). Postoperative tongue oedema was present in 5 cases (4.8%), while zero mortality was reported.

## 4. Discussion

TORS for OSA has predominantly been evaluated for its effectiveness as part of a multilevel surgical approach for moderate-to-severe OSA in patients with intolerance or CPAP failure. This is the first systematic review aiming to comprehensively evaluate objective and subjective postoperative outcome measures following TORS performed as a single-level approach in appropriately selected cases.

Overall, our review supports current evidence that single-level TORS BOT can yield clinically meaningful improvements in objective sleep outcomes, as confirmed by polysomnography. Combined mean preoperative AHI was improved significantly postoperatively from 34.2 events/hour to 14.7 events/hour, with postoperative absolute reported AHI values typically falling within the mild-to-moderate OSA range. These results are in line with previous studies, which mainly evaluated TORS as part of a multilevel approach [14,15]. However, as outlined in the study’s limitations, these findings should be interpreted with caution due to a lack of controlled comparisons, heterogeneity in follow-up duration, and limited evidence regarding long-term durability.

Evaluating TORS efficacy depends not only on mean AHI reduction, but also on the proportion of patients achieving surgical response. Surgical success was inconsistently presented across cohorts. Where Sher’s criteria were used, success ranged widely (54.2–100%), restricting accurate estimation of overall clinical effectiveness, with a weighted estimated success rate of 66.4%. This variability likely reflects differences in baseline OSA severity, selection thresholds, collapse patterns, and follow-up duration. A recent meta-analysis aimed to address this gap in the literature by performing subgroup analysis for the single-level TORS BOT procedures, concluding that the mean success rate ranged from 68% to 69% [16]. Moreover, success and cure rates represent distinct endpoints and cure, defined as postoperative AHI < 5 events/hour, was reported less frequently, underscoring that many patients experience substantial improvement yet may retain residual OSA requiring further treatment.

Regarding subjective postoperative sleep-related outcomes, patient-reported outcomes also seem to be improved; however, inconsistent reporting limits data quality and interpretation. In studies providing absolute ESS values, the combined mean decreased from 13 preoperatively to 4.5 postoperatively, suggesting marked improvement in daytime sleepiness. Nevertheless, PROM collection was heterogeneous and often limited to ESS, which primarily reflects somnolence and does not comprehensively evaluate patient-centred benefits following tongue base surgery with variable inclusion of disease-specific quality-of-life outcomes. Domains such as snoring-related burden, work performance, functional outcomes, swallow impairment and speech-related symptoms were rarely quantified using validated PROMs. This is a clinically important gap because AHI alone does not fully capture treatment benefit; sleepiness, functional status, and quality of life are central to shared decision-making and should be prioritised in future studies through standardised PROM reporting.

Operative reporting of tongue base excised tissue volume remains another limitation of the current evidence base. Only two included studies reported resection volume, and available data are insufficient to determine whether a greater extent of resection translates into larger reductions in AHI, higher rates of cure, or increased morbidity. This relationship is clinically important, as under-resection risks inadequate benefit, whereas more extensive resection may increase rates of dysphagia, bleeding, or taste disturbance. A minimum excision of 7 mL of lingual tonsil tissue had been previously reported as effective, whereas removal of >50 mL was associated with a higher risk of surgical complications [10,17,18]. Systematic reporting of resection volume metrics alongside standardised functional outcomes would strengthen interpretability, optimise surgical technique and aid comparisons with other available surgical approaches.

TORS BOT with or without concomitant EGP is considered a safe approach, with most associated adverse events being minor and self-limiting. The existing literature frequently reports complications as overall rates for heterogeneous cohorts that include both multilevel and standalone TORS procedures, limiting conclusions regarding morbidity attributable to isolated BOT/EGP surgery. Prior systematic reviews suggest that swallowing complications were more frequent in the multilevel approaches, taste complications were more common in standalone TORS, while postoperative bleeding occurred more frequently in the standalone TORS, but was typically self-limited [5,18]. Consistent with these findings, dysgeusia was the most frequent complication in our review (15.2%), and postoperative bleeding was encountered in 9.5% of cases, with 90% managed conservatively, supporting an overall acceptable safety profile for standalone TORS.

Overall, patient selection remains key in interpreting and optimising outcomes. While included studies generally aimed to enrol CPAP-intolerant patients with moderate-to-severe OSA and BMI < 35 kg/m^2^, DISE utilisation and reporting were limited. The concept of tailored treatment is particularly relevant given that epiglottic collapse can be addressed with dedicated epiglottic procedures, including standalone approaches reported with encouraging outcomes [19]. DISE classification reporting would enable more informative subgroup analyses and facilitate identification of phenotypes most likely to respond to BOT reduction alone versus BOT plus epiglottic surgery. In addition, baseline characteristics play a crucial role in surgical outcomes. The included cohorts were predominantly male (81.9%), consistent with the recognised high male predominance in OSA, ranging from 2:1 to even 8:1 in the clinical setting [20]. However, this potentially limits the generalizability of findings to female patients, while sex-stratified outcomes were not reported. Age-stratified outcomes were also missing, limiting conclusions regarding the potential influence of age-related reductions in upper airway tissue compliance and increased comorbidity burden on surgical response, postoperative recovery and healing.

A variety of non-robotic surgical approaches to address tongue base obstruction exist, and although several studies have attempted to compare these approaches with TORS, evidence remains inconclusive, with limited robust comparative effectiveness data. A recent meta-analysis aiming to assess the safety of tongue base procedures for sleep apnoea concluded that data were very heterogeneous and limited to safely draw any conclusions in terms of the superiority of surgical techniques [21]. A recent study compared Radiofrequency Tongue Ablation (RFTA) with TORS and reported significant postoperative improvement in AHI, minimum arterial oxygen saturation and ESS scores in the TORS group, with comparable complications [22]. Hwang et al. [23] reported that coblation and TORS achieved similar outcomes and comparable complication rates within a multilevel framework, while Lan et al. [24] similarly concluded that TORS achieved comparable outcomes to coblation-assisted tongue base reduction. Collectively, these findings reinforce that treatment selection should be individualised, while highlighting that meaningful comparisons across modalities are currently limited by heterogeneity in selection criteria, baseline characteristics, follow-up duration and inconsistent reporting of surgical success rates and PROMs. Consequently, current comparative studies do not permit reliable comparisons or generalisation of findings regarding the superiority of TORS over alternative techniques.

Study limitations

This systematic review carries out certain limitations, mainly stemming from the nature and limited number of available studies. The evidence remains limited and largely observational, with inherent susceptibility to selection bias and confounding. Heterogeneity across studies mainly is due to inconsistency in baseline disease severity, follow-up duration and outcome definitions. Incomplete outcome reporting, significant variation in reported surgical success rates and limited description of missing data handling also introduce bias, limiting confidence in concluding the overall efficacy estimation. As a result, the observed postoperative improvements should be interpreted as associations in carefully selected cohorts rather than definitive estimates of treatment effect. Publication bias due to surgical publications cannot be excluded, while the English-language restriction may have omitted relevant data.

Despite these limitations arising from observational designs, heterogeneity and inconsistent reporting, findings are directionally consistent, and this study successfully addresses the current literature, clarifying expected improvements in AHI and patient symptoms following single-level TORS BOT in carefully selected cases. Also, it highlights current gaps in outcome reporting, both objective and subjective. Future prospective multicentre cohorts using standardised DISE classification, objective outcomes and predefined follow-up interval are needed to define predictors of success and cure, quantifying durability of treatment benefit, and ultimately comparing single-level TORS BOT with alternative tongue base surgeries.

## 5. Conclusions

Single-level TORS BOT, with or without concurrent epiglottic surgery, appears to provide significant improvements in objective sleep outcomes and symptom burden in carefully selected adults intolerant to CPAP with moderate-to-severe OSA. Postoperative AHI reduction to mild-to-moderate ranges and ESS improvement were consistently reported among included studies, while associated morbidity was low, with most complications resolving with conservative measures. However, the generalisability of these findings should be interpreted cautiously due to the observational design, lack of standardised follow-up, heterogeneity and moderate confounding risk of included studies. Regarding subjective PROMs, interpretation was limited due to inconsistent reporting. Further studies with standardised objective and subjective outcome measures and predefined follow-up intervals are required to aid comparative evaluation of single-level TORS to other alternative surgical treatments aiming to address tongue base obstruction.

## Figures and Tables

**Table 1:** Baseline characteristics of included studies.

First Author	Year	Number of Patients	Study Design	Inclusion Criteria	Mean Follow-Up (Months)	Minimum (Range) Follow-Up (Months)
Arora [9]	2016	14	Prospective	1, 2, 3a, 4	18.9	12 (12–24)
Karaman [10]	2017	20	Prospective	1, 2	12	6 (N/A)
Huntley [11]	2018	24	Retrospective	1, 2	N/A	>1 (N/A)
Babademez [12]	2019	37	Prospective	1, 2, 3a, 4	N/A	6 (N/A)
Chekkoury Idrissi [13]	2021	10	Prospective	1, 2, 3b	N/A	12 (N/A)

1. Moderate-to-severe OSA confirmed by sleep study (AHI ≥ 15 episodes/h); 2. Failure to tolerate or comply with other OSA treatment modalities, including CPAP and mandibular advancement device (MAD); 3a. BMI < 35 kg/m^2^; 3b. BMI < 30 kg/m^2^; 4. Predominant BOT collapse with or without epiglottic collapse evaluated by DISE. Abbreviations: N/A = data not available.

**Table 2:** Objective outcome measures.

First Author	Preoperative AHI (Mean ± SD)	Postoperative AHI (Mean ± SD)	Preoperative LSAT	Postoperative LSAT	Preoperative Oxygen Saturation (Mean ± SD)	Postoperative Oxygen Saturation (Mean ± SD)
Arora	36.3 ± 21.4	21.2 ± 24.6	N/A	N/A	92.9 ± 1.8	94.3 ± 2.5
Karaman	36.2	8.8	N/A	N/A	N/A	N/A
Huntley	35.7	20.1	80.5	84.1	N/A	N/A
Babademez	29.7 ± 9	10.7 ± 3.9	N/A	N/A	N/A	N/A
Chekkoury Idrissi	39.8 ± 22.7	19.1 ± 14.2	N/A	N/A	N/A	N/A

Reported outcomes: AHI measured in episodes/hour, oxygen saturation measured in %. Abbreviations: AHI = apnoea-hypopnoea Index, LSAT = lowest oxygen saturation, SD = standard deviation, N/A = data not available.

**Table 3:** Subjective outcome measures.

First Author (Year)	Preoperative ESS (mean ± SD)	Postoperative ESS (mean ± SD)	Postoperative ESS Reduction	Snoring VAS Reduction Rate
Arora (2016) [9]	14.9 ± 5	<4	N/A	Available
Karaman (2017) [10]	N/A	N/A	N/A	N/A
Huntley (2018) [11]	N/A	N/A	N/A	N/A
Babademez (2019) [12]	N/A	N/A	33.8%	38.6%
Chekkoury Idrissi (2021) [13]	13.8 ± 5.8	5.8 ± 3.5	N/A	N/A

Normal daytime sleepiness was defined as ESS ≤ 10. Abbreviations: ESS = Epworth Sleepiness Score, VAS = Visual Analogue Scale, SD = standard deviation, N/A = data not available.

## Data Availability

Data were gathered in pre-formed Excel spreadsheets and will be made available on reasonable request.
